# Impact of DBD Plasma Jet Treatment on the Enamel Surface of Primary Teeth

**DOI:** 10.3390/ma17215173

**Published:** 2024-10-24

**Authors:** Michał Kwiatkowski, Joanna Pawłat, Agnieszka Starek-Wójcicka, Marta Krajewska, Piotr Terebun, Dawid Zarzeczny, Monika Machoy, Agnieszka Mazur-Lesz, Narumol Matsuyama, Tomoyuki Murakami, Nobuya Hayashi, Elżbieta Grządka

**Affiliations:** 1 Department of Electrical Engineering and Smart Technologies, Lublin University of Technology, Nadbystrzycka Street 38A, 20-618 Lublin, Poland; m.kwiatkowski@pollub.pl (M.K.); j.pawlat@pollub.pl (J.P.); p.terebun@pollub.pl (P.T.); d.zarzeczny@pollub.pl (D.Z.); 2 Department of Biological Bases of Food and Feed Technologies, Faculty of Production Engineering, University of Life Sciences in Lublin, 20-612 Lublin, Poland; agnieszka.starek@up.lublin.pl (A.S.-W.); marta.krajewska@up.lublin.pl (M.K.); 3 Department of Periodontology, Faculty of Medicine and Dentistry, Pomeranian Medical University, Powstańców Wielkopolskich 72 Street, SPSK 2, 70-111 Szczecin, Poland; monika.machoy@pum.edu.pl; 4 Private Dental Office, Witkiewicza 49u/14 Street, 71-124 Szczecin, Poland; abmazur@wp.pl; 5 Faculty of Science and Engineering, Saga University, Saga 840-8502, Japan; si4430@cc.saga-u.ac.jp; 6 Faculty of Science and Technology, Seikei University, 3-3-1 Kichijoji-Kitamachi, Musashino, Tokyo 180-8633, Japan; tomo-murakami@st.seikei.ac.jp; 7 Interdisciplinary Graduate School of Engineering Sciences, Kyushu University, Fukuoka 816-8580, Japan; hayashi.nobuya.056@m.kyushu-u.ac.jp; 8 Faculty of Chemistry, Institute of Chemical Sciences, Maria Curie-Skłodowska University, M. Skłodowskiej-Curie 3 Sq., 20-031 Lublin, Poland

**Keywords:** enamel surface, cold atmospheric plasma, dielectric barrier discharge, surface properties, primary teeth

## Abstract

The impact of cold atmospheric plasma (CAP) treatment on the enamel of twelve primary teeth (incisors, canines, and molars) collected from six children was examined in order to evaluate the possibility of using the CAP technique in dental applications. A radio-frequency dielectric barrier discharge (DBD) plasma jet operating at a voltage of 3.25 kV using a mixture of helium and oxygen as the working gas was used for the generation of plasma as part of the electro-technological method for the treatment of biological material. The plasma exposure time for the primary teeth was 5, 10, and 20 min. The properties of tooth enamel (color, contact angles, surface roughness, surface topography, elemental composition) were examined before (control) and after the plasma treatment. As shown by the results, the plasma treatment time is a key parameter that can induce desired features, such as whitening or improved wettability. However, with prolonged plasma treatment (20 min), the enamel surface may be permanently damaged. The cold-plasma-treated samples were characterized by a higher value of the brightness L* parameter and thus a lighter color, compared to the CAP-untreated teeth. It was also evidenced that the plasma treatment increased the hydrophilicity of tooth surfaces, and the contact angles effectively decreased with the time of the CAP treatment. The tooth surface also became much more heterogeneous and rough with much greater amplitudes in heights. The surface of the primary teeth after the CAP treatment lost its homogeneity, as evidenced by the SEM micrographs. The analysis of the elemental composition revealed only minor changes after the plasma process, which may suggest that the observed morphological changes in the enamel surface are mainly physical and are not a consequence of chemical reactions between the enamel and the reactive components of the cold plasma. Plasma treatment of teeth opens up new possibilities of using this method as an alternative to whitening or pre-treatment before other dental procedures.

## 1. Introduction

The tooth is one of the most individual and complex anatomical and histological structures in the human body. Humans exhibit diphyodonty, i.e., two sets of teeth: (i) primary and (ii) permanent teeth (Table 1). There are many differences between deciduous and permanent dentition, e.g., in the color, shape, size, and positioning of teeth in the jaws. The most important dissimilarity is the size and thickness of layers that form a tooth. It has an extreme impact on the choice of different therapeutic/treatment methods, which can be used in pedodontics. Both permanent and primary teeth consist of the same tissues, i.e., enamel, dentin, cementum, and pulp [1]. The first three are mineralized and contain mainly inorganic compounds, which is why they are called hard dental tissues. The most superficial layer, which protects the tooth and covers the entire crown, i.e., the enamel, is composed of enamel prisms and an interprismatic substance.

As for its chemical composition, the enamel contains mainly inorganic compounds, but also water and small amounts of organic substances. The main building blocks of enamel are calcium phosphate hexagonal crystals (hydroxyapatite, Ca_10_(PO_4_)_6_(OH)_2_), which are most often substituted by magnesium and sodium (replacing calcium) and carbonates (in place of hydroxyl and phosphate groups) [3]. In addition to the basic mineral components of enamel, which are calcium (ca. 36–37%) and phosphorus (ca. 17%), it also contains small amounts of potassium, sodium, magnesium, and fluorine. The main organic components of enamel are the so-called enamelins—thin structures located between enamel prisms [4]. The chemical composition of tooth enamel can be changed during the development of teeth and may change due to the high chemical reactivity and the ability to exchange ions between hydroxyapatite crystals and the external environment. Although enamel is a very strong substrate, it is quite often subject to cracks and fractures; hence, research focusing on in/ex situ hydroxyapatite coatings is gaining a lot of interest [5,6]. Since the enamel surface is a stable and hard substrate covered by a natural film of organic residues and mucin from saliva, microflora attaches more easily to the surface of teeth than to the oral mucosa. A film with an ordered structure is formed on teeth, which is an excellent substrate for the adhesion of bacteria. The oral mucosa is a difficult environment to inhabit due to the constant exfoliation of the epidermis [7,8].

One innovation in therapeutic dentistry, based mainly on the fight against bacteria living on the tooth surface as difficult-to-fight biofilms that cause infections of the mouth, teeth, and gums, is cold atmospheric plasma (CAP) application. Low-temperature plasma consists of a mixture of both ionized and non-ionized molecules, free radicals, ground-state and excited atoms, reactive nitrogen and oxygen species (RONS), ozone, electrons, and UV radiation. A number of these reactive chemical molecules are responsible for damaging microbial membranes and cell walls. The disintegration of surface structures occurs as a result of the bombardment of cells with reactive plasma components (O_2_, O_3_, OH, H_2_O_2_, NO·, and NO_2_), with the most lethal effect exerted by atomic oxygen and the hydroxyl radical, which consequently results in the disruption of their continuity and cell lysis. Cold atmospheric plasma also leads to the degradation of the DNA of microorganisms, because UV rays are characterized by a high energy value and a high degree of absorbance by DNA and RNA molecules, which leads to the formation of thymine dimers and nucleoid fragmentation [9,10,11,12,13].

Since many oral diseases are infectious (periodontitis, including gingivitis or caries), the decontaminative, antimicrobial plasma treatment could replace/support/enhance conventional surgical procedures and/or antibiotic therapy [14,15]. A unique advantage of the plasma gas phase is also the elimination of microorganisms, including *Enterococcus faecalis*, from the system of tooth root canals with complex structures, such as irregularities, deltas, isthmuses, branches, and in particular dental tubules [16,17,18]. Recent literature reports [19] compared the efficacy of diode laser, CAP, and photodynamic therapy (PDT) with two photosensitizers (PSs), for the disinfection of primary mandibular second molar root canals colonized by *E. faecalis*. The treatments reduced the colony count, and, taking into account the side effects, they seem to be more acceptable in the case of children than 2.5% NaOCl. Moreover, in the case of primary dental pulpectomy, NaOCl may have a negative impact on the oral mucosa or surrounding tissues and, what is more important, on the dental follicle of permanent successors (potential root resorption) [20]. When bonding ceramic restorations, thereby improving the hydrophilicity of the surface, plasma techniques were tested as an alternative/addition to conventional procedures. Importantly, this technique does not require the use of toxic chemicals applied in this type of treatment, including etching feldspar ceramics when hydrofluoric acid is applied and a silane coupling agent is used for coating [21,22,23,24]. Ionized gas can also treat superficial tissue infections [25,26] or support tooth bleaching without thermal damage [27,28]. Claiborne et al. [27] achieved a statistically significant difference in tooth brightness with 10 and 20 min exposures to low-temperature plasma delivered using a plasma pencil in combination with 36% H_2_O_2_ gel, compared to 36% hydrogen peroxide used alone. The results reported by Yang et al. [29] also prove that the time after which a proper tooth whitening effect can be achieved (without affecting the tooth enamel and pulp tissue) is 5–10 min of plasma with air used in combination with H_2_O_2_ gel in an amount ranging from 15 to 35%.

Due to the diversity of the morphological structure of teeth, different plasma geometry parameters, different properties of plasma processors, and methods of setting and expressing process parameters, many potential applications of plasma for dentistry purposes remain unexplored. As far as we know, the impact of CAP on primary teeth has not yet been well understood. This is therefore a pioneering study undertaken to start determining the impact of CAP generated in a dielectric barrier discharge (DBD) plasmatron on the morphological structure, roughness, and chemical composition of teeth after plasma treatment. Primary teeth were selected for the research because they have thinner enamel and are less mineralized. Therefore, any negative effects (clinically significant during dental procedures) of cold plasma treatment will be more visible than in permanent teeth. Additionally, the results of research conducted on more delicate and demanding material (primary teeth) can be successfully applied to more resistant permanent teeth. The research was conducted to estimate changes in tooth enamel induced by cold atmospheric plasma and to answer the question of whether this technique can be a safe alternative to currently used methods of whitening (in permanent dentition) or increasing hydrophilicity and roughness before various dental procedures (possibly cold plasma increases the strength of bonding of dentin and enamel to selected restorative materials, which is crucial in dentistry). The hypothesis predicts that cold plasma treatment ensures a brightening effect, simultaneously destroying the enamel structure of deciduous teeth.

## 2. Materials and Methods

### 2.1. Studied Materials

Primary teeth of six children were selected as the research material. They were voluntarily donated by parents, and written consent was provided by legal guardians (parents). Biological samples, such as primary teeth that are voluntarily donated by legal guardians after the natural fallout of baby/primary teeth and are not used for genomic research, do not require approval by the Bioethical Commission. At least two teeth of the same type were collected from each child, e.g., two incisors, two canines, or two molars. The first tooth from the sample was the reference material, and the second one was exposed to low-temperature air plasma for 5 min (samples: 1-incisor, 2-incisor, 4-canine), 10 min (samples: 3-incisor, 5-molar, 9-canine), and 20 min (samples: 6-canine, 7-incisor, 10-canine). The inclusion and exclusion criteria for the primary teeth selected for this study are presented in Table 2.

### 2.2. Plasma Treatment

**Figure 1:**
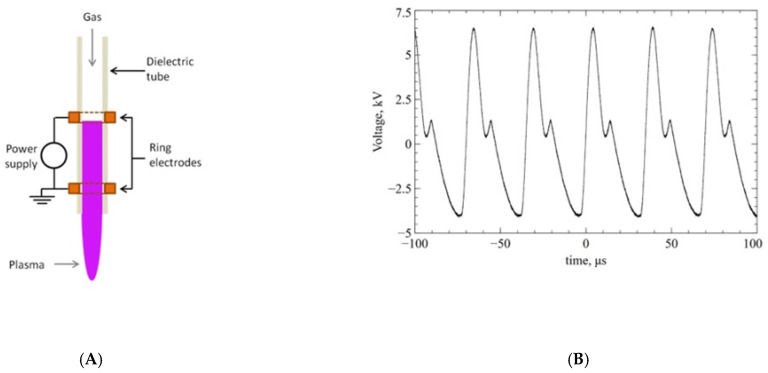
Scheme of a plasma reactor with dielectric barrier discharge. (**A**) Configuration of two electrodes; (**B**) Voltage characteristics of the power supply.

Figure 1 shows the atmospheric pressure DBD plasma jet geometry, which was already presented in our previous work [30]. The reactor consists of a glass tube (length 100 mm, diameter 5 mm) with two ring-shaped electrodes mounted outside. Due to the presence of noble gas (helium) that reduces the ignition voltage, a stable plasma volume is generated inside the tube, and the produced active particles are directed outside the reactor by a forced gas flow. The stream with active particles was directed perpendicular to the sample surface, covering the entire treated surface. Due to the limited number of available samples, only one discharge geometry and power supply parameters were chosen, assuming that time would be a key processing parameter for the reactor used [31]. The distances (10 mm from the sample to the end of the reactor, electrodes spaced 10 mm apart) were determined experimentally, for which the temperature of the processed material (a tooth fragment not suitable for surface analysis) was lower than 35 °C for all processing times (5, 10, and 20 min). The output voltage (3.25 kV RMS) waveform of the radio frequency (28.5 kHz) power supply, which confirms the stability and repeatability of the discharge, is shown in Figure 1B. The forced gas flow was regulated using direct reading glass flowmeters (Zaklady Automatyki ROTAMETR, Gliwice, Poland), with which a mixture of helium (purity 4.0, flow rate 1.667 dm^3^/min) and oxygen (purity 5.0, flow rate 0.013 dm^3^/min) was obtained. The selected flows allowed the application of the highest possible level of oxygen, which potentially could have an impact on the amount of nitrogen and oxygen particles produced.

### 2.3. Color Measurement

A spectrophotometer SV-300 (3Color, Marcq-en-Barœul, France) with the D65° illuminant with an angle of observation of 10°, with the CIELAB color space (L*a*b*) was used. The camera contained a 3 × 1 mm diaphragm. Before starting the analyses, the device was calibrated by reading the white standard and the black standard (zero calibration) and, if necessary, checking the correctness of the data reading for the white standard.

### 2.4. Water Contact Angle

The water contact angle (WCA) was measured using a DSA25E goniometer (KRÜSS, Hamburg, Germany) equipped with a CCD camera with a 73 dB dynamic range and a monochromatic (420 nm) LED as a light source. Given the intention to investigate the effect of plasma treatment in conditions simulating potential practical usage, the contact angle was tested directly on a natural uncut enamel surface. Considering the relatively small size and rounded shape, the tests of each tooth were performed only in one selected area characterized by the flattest and most extensive surface possible. Wettability was determined with the sessile drop methodology using distilled water with a volume of 1 μL dropped onto the surface using an automatic dispenser. The contact angle was calculated 1 s after placing the drop on the surface using the ellipse (tangent − 1) fitting method. For teeth before processing, four repetitions were performed at an interval of 48 h to minimize the possible change in the enamel contact angle as a consequence of the impact of water alone [32]. Only a single measurement was made in the plasma-treated samples due to the possible interference with the effect of the angle returning to its previous value, which will be discussed in greater detail in the next section. In order to test the long-term impact of the plasma, the WCA measurement was repeated 96 h after the treatment in samples left in ambient conditions at a temperature of 21 ± 2 °C.

### 2.5. SEM—EDX Analysis

The morphology of teeth (control sample) and the same samples after the plasma treatment (air plasma, time of plasma treatment: 5, 10, or 20 min) and their elemental composition (EDX) were analyzed using a scanning electron microscope (SEM, Quanta 3D FEG, Fei, Hillsboro, OR, USA) with the applied voltage of 5 kV. The photomicrographs were taken at 1000× magnification. This magnification was chosen because it allows analyzing the effect of plasma on the surfaces, but the naturally occurring morphological differences between the examined samples could be neglected. Quantitative results were averaged from three replicates, and standard deviations were reported.

### 2.6. Surface Roughness Characterization

An optical profilometer (Contour GT-K1, Bruker, Germany) was employed to study the topography of the surfaces of the teeth in the VXI measurement mode. This apparatus facilitates the assessment of the sample’s microgeometry by recording three-dimensional surface images and measurements of metrological parameters. The basis of the operation of the device is light interference and the imaging of interference fringes. The surface characterization was conducted based on the topography of each sample. Three different places on a 58 μm × 44 μm surface were topographically analyzed, and average values were reported. Parameters of the surface texture, such as waviness and roughness, were tested. The amplitude (height) parameters relative to reference planes Ra, Rp (height of the highest profile elevation), Rq (root-mean square; square mean of profile elevations), Rt (peak-to-valley difference), and Rv (value of the largest profile depression) were of the highest importance, and Veeco Vision 4.20 software was used for calculations.

## 3. Results and Discussion

### 3.1. Color Measurement

The color of human teeth is crucial, especially in aesthetic dentistry, and the innovative whitening method using cold plasma may revolutionize this clinical procedure. Owing to the use of optical emission spectroscopy, it has been evidenced that reactive oxygen species, especially singlet oxygen (^1^O_2_) and hydroxyl (*OH) radicals, are key factors in this process, and the level of tooth whitening is correlated with the CAP substrate gas type and the treatment time [33,34]. At the initial stage of the study, the impact of the CAP treatment time on the color of primary teeth was studied (Table 3). The L* parameter determining brightness was in the range of 38.63–69.14 in the tested plasma-untreated samples and from 42.11 to 70.06 in the plasma-treated samples. The lowest value was determined in a plasma-untreated tooth (1-incisor), and the highest value was determined in a tooth that was treated with plasma for 10 min (3-incisor). The a* parameter (color change from green to red) had negative values for all the samples, ranging from −3.21 to −11.78. The b* parameter (color change from blue to yellow) was negative in most cases. Positive values were obtained in the case of the following samples: 1-incisor (6.53), 2-incisor (24.78), and 6-canine (5.95) not treated with cold plasma (teeth more yellow). The method used resulted in a reduction in these values, which were −4.86, 14.90, and 4.52, respectively. The analyzed primary teeth showed differences in the brightness parameter L*, depending on their type. Importantly, however, the cold-plasma-treated samples were characterized by a higher value of the parameter and thus a lighter color, compared to the CAP-untreated teeth. The chromatic color parameters in the tested samples were negative for a* (greener than red) and negative for b* (bluer than yellow) for all the teeth. The higher values of the b* parameter in the case of the cold-plasma-untreated teeth indicate a higher share of yellow color. Choi et al. also reported a whitening effect of CAP on teeth [35]. They examined the effect of 10 min of cold plasma exposure at atmospheric pressure with various gases on external tooth whitening. As shown by the results obtained, a gas mixture consisting of air (50%) and oxygen (50%) caused quite a noticeable change in the color. Lee et al. [36] used a plasma jet with helium as a substrate gas, which was applied for 10 min and contributed to the improvement of the whitening result of hydrogen peroxide (H_2_O_2_). It was caused by the decomposition of proteins deposited on the tooth surface and enhanced *OH production by plasma. Cheng et al. [28] aimed to obtain a whitening effect in teeth subjected to a flow of helium with physiological saline, H_2_O_2_ gel, and a low temperature, an atmospheric pressure plasma stream with the helium process gas (He-APPJ), and a physiological saline solution. The whitening efficiency using He-APPJ was the highest among the three treatments. The greatest increase in brightness (L*) and the greatest decrease in redness (positive a*) and blueness (negative b*) were observed.

### 3.2. Water Contact Angle

The average WCA before the treatment had the highest values for the incisors (more than 95 degrees) and the lowest value for the canines, where it oscillated around 70 degrees in two cases (Table 4). The differences between the tooth types and individual samples may be due to their purpose and individual patient characteristics, which may affect the chemical composition and crystallinity of the enamel [37]. Due to the lack of a perfectly flat surface, small differences were also visible between the angles determined on both sides of the droplets (left and right).

**Figure 2:**
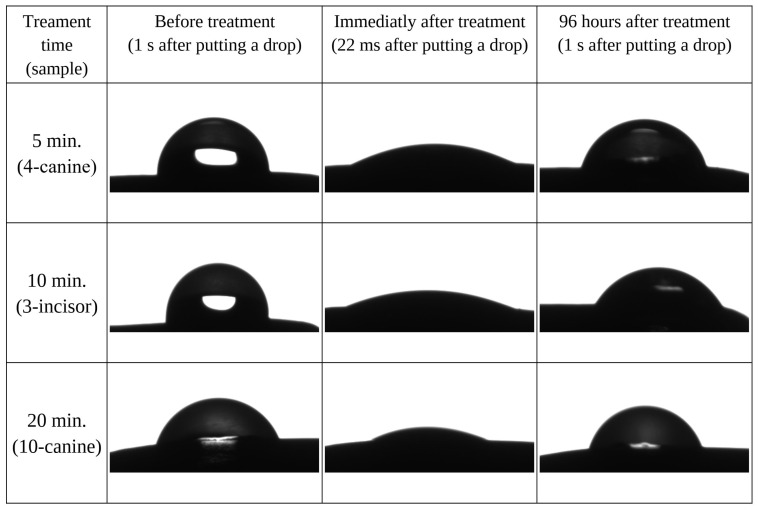
Photos of drops before and after plasma treatment.

Some examples of images before (control) and after CAP showing drops on the surface of selected teeth are presented in Figure 2.

**Figure 3:**
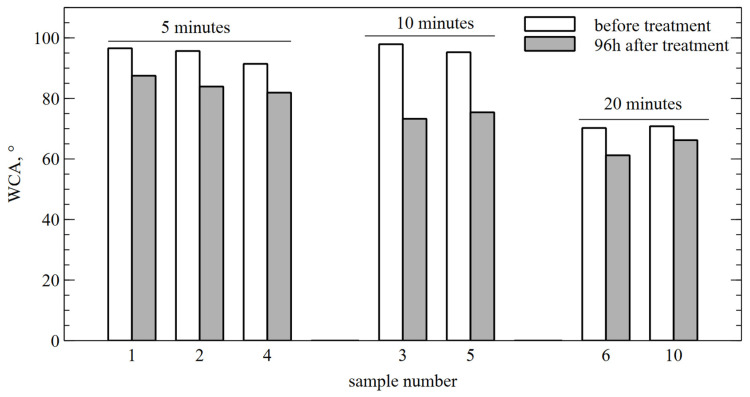
WCA of individual samples before and after CAP for selected treatment times.

Immediately after the plasma treatment, an instant spread of the drops on the tooth surface was observed for all the treatment times. Because of the significant enlargement in the area occupied by the drops conjoined with the limited area of the flat surface, the edges of the drops were hidden behind the higher parts of the tooth, which made it impossible to correctly determine the contact angle. The drop tops shown in the figure were recorded immediately after placing the drop, which spread completely on the tooth surface in less than 0.1 s. This, combined with the previously observed shape, may indicate the superhydrophilicity of the surface. After repeating the measurements after 96 h, it was again possible to observe the static position of the droplet and determine the WCA. The observed effect of recovery of surface hydrophobicity varied between individual samples (changes ranged from 6 to 25% compared to the initial value, without any noticeable trend), but in no case did it fully return to the properties observed before the treatment, which may indicate permanent changes in the material (Figure 3).

**Figure 4:**
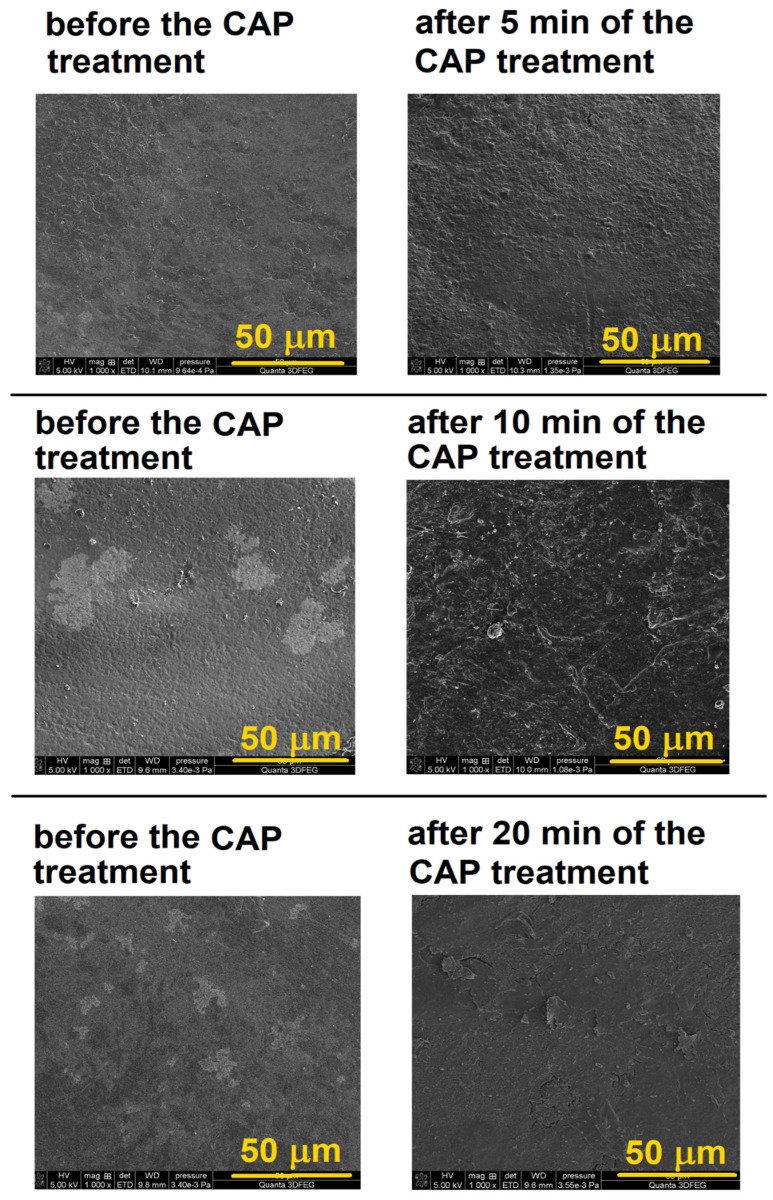
The most representative micrographs of surfaces of teeth before and after the plasma treatment.

**Figure 5:**
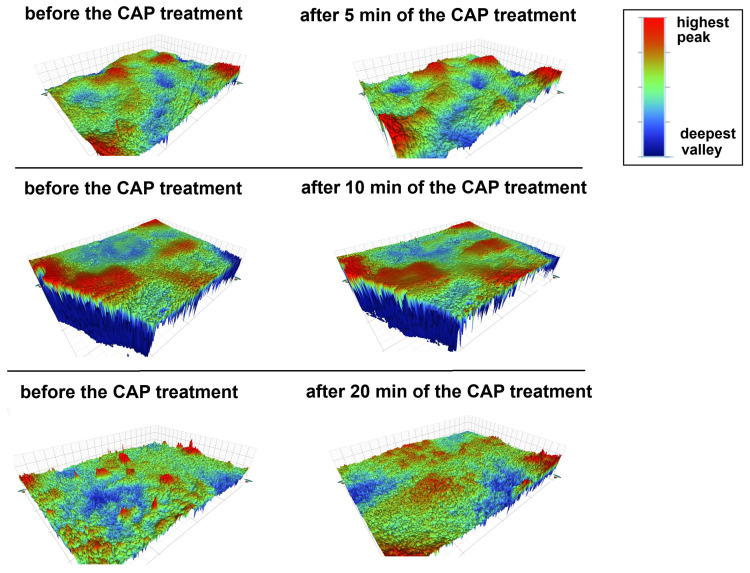
The most representative images of tooth surfaces before and after the CAP treatment (the colors represent the topography of the surface: the higher the peaks, the more red; the deeper the valleys, the more navy blue).

The phenomenon of a significant reduction in WCA after plasma treatment and its subsequent return to the initial value has already been observed by other research groups for permanent teeth. V. Šantak’s research team observed changes of 45% in dentine and 32% in enamel in relation to the initial WCA value, which then started to return to the initial value 24 h after the treatment [38]. They linked the increase in wettability to the decrease in carbon and nitrogen amounts in molecules and the formation of oxygen and hydrogen polar groups. Stasic et al. used a low-power reactor (up to 3 W) to study the change in contact angle with diiodomethane, water, and ethylene-glycol [39]. The largest change was observed in WCA (from 84.2° ± 2.6° to 6–30°), which is expected to be related to polar interactions, while excluding changes in microstructure based on SEM images. A similar explanation for this phenomenon is given by M. Chen’s team, who, on the basis of X-ray photoemission spectroscopy, related a decrease in WCA to a reduction in the content of carbon equally balanced by an oxygen content increment [40]. In turn, in addition to the influence of the presence of C=O bindings indicating hydrophilic groups on the surface, A. Lehmann’s research team highlighted the important role of changing surface roughness and surface morphology associated with the removal of organic prisms and crystallites from enamel caused by etching [41]. The results of our experiment may rather be related to a change in surface morphology, visible during both the SEM analysis and the profilometer measurements (Figure 4 and Figure 5). The noticeable change in roughness can be correlated with the change in WCA due to the etching of organic deposits and enamel substrate [41], as well as other effects related to the physical change in the tooth surface, such as capillary forces, which drag liquids from the droplet deposition spot, thus making the material super-hydrophilic [32,42,43]. This may also explain the permanent surface changes and the inability of the angle to return to its previous value, even after a relatively long time after the treatment. On the other hand, the appearance of the return effect itself must be associated with non-permanent changes, which may be related to small surface changes in the polar groups that are imperceptible to the diagnostic methods used or the effect of alteration in the surface energy value induced by the presence of charged plasma particles [32,44].

### 3.3. SEM—EDX Analysis

In order to confirm the observations presented above, SEM micrographs of the enamel surface were taken before and after plasma plating (5 min, 10 min, or 20 min) (Figure 4). The surface of primary teeth before the CAP treatment is relatively uniform topographically, although some micrographs show lighter spots that are most likely dental plaque. After a five-minute exposure to cold plasma, the tooth surface becomes more uneven, and its roughness increases noticeably. Increasing the plasma plating time to 10 min causes an even greater increase in surface heterogeneity. However, the greatest changes are observed after the 20 min exposure of the enamel to ionized gas. In such conditions, not only an increase in surface roughness and heterogeneity is observed, but there is also a loss of its continuity and the appearance of delaminated surface elements that stick out from the rest of the material. This means that the enamel surface has been damaged and indicates that the plasma plating time must be chosen with great care because too long a CAP treatment can cause serious damage and can irreversibly destroy the surface.

Based on the results presented above, the impact of CAP on the structure of the tooth enamel surface is clearly visible. However, the question arises whether bombarding the surfaces of teeth, composed mainly of hydroxyapatite, with highly reactive plasma components (e.g., O, O_2_, O_3_, OH, H_2_O_2_, NO·, and NO_2_) causes chemical changes in tooth surfaces. The composition (wt.%) of the surface enamel of primary teeth determined with the SEM-EDS quantitative analysis is shown in Table 5. Measurements were performed in preselected spots on the surface before CAP and after the plasma treatment.

The enamel of plasma-untreated primary teeth contains significant amounts of oxygen (29.94 ± 4.2–35.50 ± 3.4), calcium (31.72 ± 3.30–39.00 ± 3.95), phosphorus (15.40 ± 1.62–18.40 ± 1.2), carbon (17.92 ± 2.22–7.02 ± 0.58), and nitrogen (4.09 ± 0.33–4.13 ± 0.22) which is in agreement with the literature [45,46,47]. The other elements detected in the sample: fluorine, sodium, magnesium, potassium, and chlorine are in amounts below one percent by weight. Taking into account the enamel composition, which is made of hydroxyapatite hexagonal crystals (Ca_10_(PO_4_)_6_(OH)_2_), the high content of oxygen, calcium, and phosphorus is fully understandable. Oxygen is the main element found in hydroxyapatite as well as in water and organic compounds that build enamel [48]. Calcium and phosphorus also build blocks of hydroxyapatite. The larger content of carbon and nitrogen in the enamel of primary teeth compared to their percentage in the elemental composition of permanent teeth probably results from the larger interprismatic space in primary teeth and is a consequence of the greater porosity and permeability of primary teeth [1]. This phenomenon directly translates into the higher content of the above-mentioned elements. The analysis of the data allows the conclusion that there are several percent differences between teeth obtained from different patients, resulting from individual characteristics and their diet. For example, a higher percentage of phosphorus is a consequence of the protein substructure of enamel (amelogenin, enamel, and ameloblastin) due to phosphorylation during the calcification process [49]. However, higher carbon content may be a result of the diet when organic matter affects the enamel surface [46]. The comparison between the percentage compositions of the enamel of the plasma-treated primary teeth shows that the differences obtained have no statistical significance. This means that the morphological changes in the enamel surface (Figure 4) are mainly physical and are not a consequence of chemical reactions between the enamel and the reactive components of the cold plasma. This observation is highly relevant because the absence of significant changes in the chemical composition during the tooth plasma plating process can make the process significantly more attractive. Moreover, the Ca/P ratio representing the mineralization of hard tissues is the same before and after the CAP treatment, which indicates that CAP does not deteriorate the quality of hydroxyapatite.

### 3.4. Surface Roughness Characterization

Characterization of surface properties using profilometry is not only interesting but also very important because the roughness of a surface influences its mechanical behavior and affects the strength of materials [43,50,51]. The impact of CAP and the plasma treatment time on the enamel surface of primary teeth (Figure 5) was examined, along with parameters characterizing the surface texture, mainly focusing on waviness and roughness (Table 6). The colored bar next to the map indicates the scale of changes in the scanning depth. Blue indicates locations below the reference plane, and red indicates locations above this plane.

A comparative analysis of the presented figures clearly shows that the enamel surface changes under the influence of low-temperature plasma treatment. The surface becomes much more heterogeneous and rough with much greater amplitudes in heights. The part of Figure 5 corresponding to the comparison of the enamel surface before the plasma treatment and after the 5 min plasma exposure clearly shows the deepening of previously existing valleys (more blue color) and the enlargement of the existing hills (more red color). A tooth exposed to plasma for 10 min also changes the surface topography. It can be observed that the hills present on the right side are increasing. The greatest impact of low-temperature plasma was noticed after 20 min of exposure to ionized gas. In this case, the surface changes significantly. Small, relatively steep hills disappear under the influence of the plasma and are replaced by a larger area containing lower hills. All observed changes are the result of bombarding the surface with ionized gas, which causes morphological changes in the tooth enamel. The above analysis can also be supported by mathematical parameters (Table 6). The comparison of the magnitudes describing the topography of the tooth surfaces (Ra, Rp, Rq, Rt, and Rv) before and after the plasma plating process clearly indicates increasing roughness of these surfaces (increase in Ra), increasingly higher hills (increase in Rp and Rq), increasingly deeper valleys (increasingly negative Rv), and an increasingly larger difference between the peaks and the valleys (larger Rt value). Additionally, by analyzing the impact of the CAP treatment time on the topographic parameters of the surface before and after this process, it can be concluded that, with the increase in time, the observed differences in the magnitudes describing the topography of the surfaces become larger, which means that the longer the exposure to cold plasma, the more the enamel surface changes.

## 4. Conclusions

The study results show that the research hypothesis was partially true. The CAP application on the enamel of deciduous teeth had a brightening effect, but the destruction of the dental surface depended on the time of exposure. The cold-plasma-treated samples were characterized by a higher value of the L* parameter and thus a lighter color, compared to the CAP-untreated teeth. The chromatic color parameters in the tested samples were negative for a* (greener than red) and negative for b* (bluer than yellow) for all the teeth. The higher values of the b* parameter in the case of the cold-plasma-untreated teeth indicate a higher share of yellow color. It was also evidenced that the plasma treatment increased the hydrophilicity of tooth surfaces, and the contact angles effectively decreased with the time of the CAP treatment. The tooth surface also became much more heterogeneous and rough with much greater amplitudes in heights, and the longer the plating time, the greater the above-mentioned effects. The surface of the primary teeth after the CAP treatment lost its homogeneity, as evidenced by the SEM micrographs. Interestingly, the analysis of the elemental composition revealed only minor changes after the plasma treatment, which may suggest that the observed morphological changes in the enamel surface are mainly physical and are not a consequence of chemical reactions between the enamel and the reactive components of the cold plasma. Most likely, these processes result from the effective bombardment of the tooth surface by ionized and non-ionized molecules, free radicals, and ground-state and excited atoms, whose action is intensified by ultraviolet radiation. As an experimental outcome, it can be concluded that the time between 5 and 10 min is optimal for whitening teeth and increasing their hydrophilicity and roughness without damaging the enamel structure.

Due to the limited number of samples (the limited group of the primary teeth studied resulting from the quite restrictive inclusion and exclusion parameters of collection of teeth and the necessity to obtain teeth immediately after falling out), the present investigations are preliminary, but the results give high hopes for the use of the technology in practice. Future research should include the use of a larger number of samples for a more complete statistical analysis and in vivo studies that will also take into account the unique oral microflora. Another idea is to obtain primary teeth after trauma from aseptic necrosis of the pulp or from children suffering from hemolytic diseases in order to test the possibility of lightening these teeth using cold plasma. An important limitation here is the need to use the reactor directly during the treatment. This problem could be solved by developing a flexible extension material for indirect treatment, which would allow the transport of active plasma particles while eliminating the danger of high voltage.

## Figures and Tables

**Table 1:** Main differences between primary and permanent teeth (based on [2]).

Primary Teeth	Permanent Teeth
Number: 20 (without premolars, 3rd molars).	Number: 32.
Size/color: Smaller size/crown dimensions, bluish white (milk white). Presence of neonatal lines (enamel/dentin).	Larger in overall dimensions in comparison to milk teeth, yellowish white, grayish white. Neonatal lines in the 1st molar only.
Lower mineralization and density of enamel and dentin.	Enamel and dentin are more mineralized.
Shape: Mesiodistal dimension is larger than the height of the crown.	Cervico-incisal height dominates over the width in mesiodistal dimension (longer).
Root: Primary molars are weaker with longer, slender roots and a wider crown. Primary roots undergo physiologic resorption, and primary teeth are shed naturally.	Stronger, shorter, and more voluminous (in comparison to the crown size) permanent roots are firmly docked in jawbone. Physiologic resorption is absent.
Pulp: Pulp chambers of deciduous teeth are proportionately larger compared to the crown size. Pulp horns are higher and closer to the surface of the tooth. The pulpal outline of the primary tooth follows DEJ (dentin–enamel junction) more closely than that of permanent teeth.	Pulp chamber is smaller in relation to the crown size. The pulp outline follows DEJ less closely. Pulp horns are lower and away from the dental surface.
Root canals: Multiple ramifications, ribbon-like shape, and branching paths, which make debridement and root canal treatment very difficult.	Better defined shape, less branching.
Enamel: thinner, about 1 mm.	Up to 2.5–3 mm.
Cementum: thinner, with the presence of primary cementum only.	Thicker, with the presence of primary and secondary cementum.

**Table 2:** Inclusion and exclusion criteria for primary teeth selected for the present study.

Inclusion Criteria	Exclusion Criteria
Clinical examination confirmed the absence of dental caries or restored cavities	Dental caries at any stage (initial caries, medium caries, profound caries), presence of filling material
Parents and the patient deny previous trauma around the collected tooth	Previous dental trauma around the collected tooth confirmed by parents and the patient
Clinical examination and patient interview exclude medical conditions that could result in discoloration of primary teeth (e.g., hemolytic disease of newborns, amelogenesis imperfecta, dentinogenesis imperfecta, prematurity)	Discoloration of teeth
Obvious signs of physiological resorption of dental roots	Teeth without signs of physiological resorption of dental roots, which were extracted because of orthodontic indications
Healthy children without chronic diseases who do not take any medications permanently could take part in the study	Children with chronic diseases, taking medications permanently

**Table 3:** Impact of CAP on tooth color parameters (*n* = 3, mean ± SD).

Tooth Type	Treatment Time (min.)	Coloring Index		
		L*	a*	b*
1-incisor	0	38.63 ± 0.03	−9.31 ± 0.01	6.53 ± 0.04
2-incisor	0	40.58 ± 0.37	−3.51 ± 0.02	24.78 ± 0.46
3-incisor	0	60.02 ± 0.94	−6.45 ± 0.02	−1.74 ± 0.39
4-canine	0	69.14 ± 0.02	−5.70 ± 0.00	−2.19 ± 0.01
5-molar	0	40.98 ± 0.26	−11.78 ± 0.08	−1.63 ± 0.54
6-canine	0	54.72 ± 0.15	−3.21 ± 0.01	5.95 ± 0.08
7-canine	0	57.16 ± 0.01	−5.85 ± 0.01	−0.95 ± 0.01
1-incisor	5	42.11 ± 0.01	−10.21 ± 0.01	−4.86 ± 0.01
2-incisor	5	40.83 ± 0.38	−5.17 ± 0.04	14.90 ± 0.12
3-incisor	10	60.29 ± 0.08	−6.25 ± 0.02	−1.23 ± 0.01
4-canine	5	70.06 ± 0.16	−6.00 ± 0.00	−3.86 ± 0.01
5-molar	10	48.65 ± 0.01	−10.25 ± 0.00	−4.69 ± 0.11
6-canine	20	53.32 ± 0.15	−5.08 ± 0.03	4.52 ± 0.13
7-canine	20	60.15 ± 0.01	−7.49 ± 0.01	−8.29 ± 0.01

**Table 4:** WCA in samples before plasma treatment (mean ± SD).

Tooth Type	WCA (Left)	WCA (Right)	WCA (Mean)
1-incisor	96.32 ± 1.57	96.81 ± 1.59	96.57 ± 1.58
2-incisor	95.76 ± 2.60	95.55 ± 2.78	95.66 ± 2.67
3-incisor	97.87 ± 3.80	97.93 ± 4.28	97.90 ± 3.99
4-canine	91.04 ± 2.40	91.80 ± 1.19	91.42 ± 1.78
5-molar	94.52 ± 2.98	96.01 ± 2.46	95.26 ± 2.69
6-canine	69.06 ± 5.42	71.41 ± 5.21	70.23 ± 5.30
10-canine	70.96 ± 3.41	70.65 ± 2.30	70.80 ± 2.84

**Table 5:** Surface composition (wt.%) of the enamel of primary teeth before and after CAP.

	Before the CAP Treatment	After 5 min of the CAP Treatment	Before the CAP Treatment	After 10 min of the CAP Treatment	Before the CAP Treatment	After 20 min of the CAP Treatment
O	35.50 ± 3.47	37.58 ± 2.81	29.97 ± 3.06	28.92 ± 2.11	29.94 ± 4.25	29.28 ± 2.87
Ca	35.11 ± 5.69	35.00 ± 4.93	39.00 ± 3.95	39.12 ± 2.58	31.72 ± 3.30	33.00 ± 2.46
P	17.01 ± 1.85	16.40 ± 1.96	18.40 ± 1.24	18.31 ± 1.56	15.40 ± 1.62	16.40 ± 2.04
C	7.02 ± 0.58	5.39 ± 1.01	7.55 ± 0.51	6.30 ± 0.59	17.92 ± 2.22	16.39 ± 1.15
N	4.09 ± 0.33	4.20 ± 0.26	4.13 ± 0.22	6.23 ± 0.44	4.12 ± 0.57	3.20 ± 0.02
F	0.46 ± 0.03	0.49 ± 0.04	0.12 ± 0.01	0.13 ± 0.01	0.04 ± 0.01	0.79 ± 0.05
Na	0.52 ± 0.02	0.65 ± 0.04	0.33 ± 0.02	0.64 ± 0.05	0.53 ± 0.04	0.65 ± 0.04
Mg	0.12 ± 0.02	0.09 ± 0.01	0.15 ± 0.02	0.17 ± 0.02	0.17 ± 0.01	0.09 ± 0.03
K	0.07 ± 0.01	0.09 ± 0.03	0.08 ± 0.01	0.06 ± 0.03	0.06 ± 0.02	0.09 ± 0.02
Cl	0.10 ± 0.01	0.11 ± 0.01	0.27 ± 0.02	0.12 ± 0.04	0.1 ± 0.01	0.11 ± 0.02
Ca/P	2.06	2.13	2.12	2.14	2.06	2.01

**Table 6:** Roughness parameters of primary teeth before and after the CAP treatment. SD ≤ 8%.

**1-Incisor**	**Before CAP**	**After 5 min of CAP**	**Difference**
Ra	1.12	1.34	0.22
Rp	7.01	11.08	4.07
Rq	1.46	1.70	0.24
Rt	12.56	20.03	7.47
Rv	−5.55	−8.96	−3.41
**3-Incisor**	**Before CAP**	**After 10 min of CAP**	**Difference**
Ra	1.05	1.28	0.23
Rp	5.99	6.93	0.94
Rq	1.77	2.49	0.72
Rt	32.95	46.24	13.29
Rv	−26.96	−39.31	−12.35
**7-Canine**	**Before CAP**	**After 20 min of CAP**	**Difference**
Ra	1.70	2.04	0.34
Rp	10.83	19.09	8.26
Rq	2.96	4.49	1.53
Rt	34.26	69.95	35.69
Rv	−23.43	−50.86	−27.43

## Data Availability

Data are contained within the article.
